# 气相色谱-三重四极杆质谱法同时测定纺织品中11种挥发性全氟化合物前体物

**DOI:** 10.3724/SP.J.1123.2021.01043

**Published:** 2021-11-08

**Authors:** Chunlan WANG, Haixuan ZHANG, Li ZHU, Wangxia HU, Ziwei LIN

**Affiliations:** 1.深圳市计量质量检测研究院, 广东 深圳 518109; 1. Shenzhen Academy of Metrology and Quality Inspection, Shenzhen 518109, China; 2.广州市疾病预防控制中心, 广东 广州 510440; 2. Guangzhou Center for Disease Control and Prevention, Guangzhou 510440, China

**Keywords:** 气相色谱-串联质谱法, 全氟化合物前体物, 纺织品, gas chromatography-tandem mass spectrometry (GC-MS/MS), perfluorinated compound precursors, textiles

## Abstract

以甲醇为提取溶剂,超声辅助提取纺织品中的全氟化合物前体物,建立了一种气相色谱-三重四极杆质谱(GC-MS/MS)法同时测定纺织品中11种挥发性全氟化合物前体物:4种氟调聚物醇(FTOHs)、3种氟调聚丙烯酸酯(FTAs)、2种全氟辛基磺酰胺(FOSAs)和2种全氟辛基磺酰胺乙醇(FOSEs)。考察了超声提取溶剂、提取温度和提取时间对提取效率的影响,最终确定用甲醇为提取溶剂,70 ℃下超声提取60 min,目标物经VF-WAXms毛细管柱(30 m×0.25 mm×0.25 μm)程序升温分离,GC-MS/MS多反应监测(MRM)模式检测,外标法定量。实验结果表明:11种挥发性全氟化合物前体物在10~500 μg/L范围内线性关系良好,相关系数(*r*)均不低于0.9984;以信噪比为3计算,检出限(LOD)为0.002~0.04 mg/kg;以信噪比为10计算,定量限(LOQ)为0.006~0.1 mg/kg;不同材质纺织品中,11种挥发性全氟化合物前体物在高、中、低3个添加水平下的回收率为73.2%~117.2%,相对标准偏差(RSD)为0.1%~9.4%(*n*=6)。该方法前处理简单,定性、定量准确,灵敏度高,重现性好,可有效用于纺织品中11种挥发性全氟化合物前体物的同时检测。实际样品分析发现,当前全氟化合物前体物已被应用于纺织品整理当中。该方法的建立对我国纺织品中全氟化合物前体物风险物质的管控和检测标准的制定具有一定的理论和现实意义。

## Abstract (en)

Perfluorinated compounds (PFCs) are persistent organic compounds. PFCs are artificially prepared hydrocarbons in which hydrogen atoms are completely replaced by fluorine. PFCs have excellent thermal stability and chemical stability, high surface activity, and hydrophobic and/or oleophobic properties owing to their exceptionally strong C-F bonds, low polarizability, and weak intermolecular van der Waals interactions. Currently, PFCs and their precursors are widely used in textile production as finishing agents and surfactants. In recent years, increasing attention has been devoted to PFCs and their precursors. In many countries and regions, such as the European Union, Canada, Denmark, and the United States, directives and regulations have been issued to restrict the use of PFCs and their precursors; the number of these compounds in such lists is increasing continuously. Studies have shown that PFCs are hepatotoxic, embryotoxic, reproductive-toxic, neurotoxic, and carcinogenic, and can interfere with the endocrine system, change animal instinct behavior, and potentially induce developmental neurotoxicity in humans, especially in young children. However, there are few established methods for the simultaneous detection of multiple PFC precursors, necessitating the same particularly for textiles. In this study, a method was developed for the simultaneous determination of 11 volatile PFC precursors in textiles using gas chromatography-triple quadrupole tandem mass spectrometry (GC-MS/MS). The target compounds included four fluorotelomer alcohols (FTOHs), three fluorotelomer acrylates (FTAs), two fluorooctane sulfonamides (FOSAs), and two fluorooctane sulfonamide-ethanols (FOSEs). Studies have shown that FTOHs and FTAs are precursors of perfluorocarboxylic acid, and FOSAs are precursors of perfluorooctanesulfonic acid. Some PFC precursors are converted into perfluorocarboxylic acid and perfluoroalkyl sulfonic acid, which threaten human health and ecological security. In this study, an effective ultrasonic-assisted extraction method for the 11 target compounds was established. The effects of the extraction solvent, extraction temperature, and extraction time on the extraction efficiency were investigated. The optimum extraction conditions for the developed method were carrying out ultrasonic extraction at 70 ℃ for 60 min with methanol as the extraction solvent. Separation was performed on a VF-WAXms capillary column (30 m×0.25 mm×0.25 μm) with temperature programming, following which the target compounds were detected by GC-MS/MS in the multiple reaction monitoring (MRM) mode and quantified using the external standard method. The matrix effects of three textile matrices were also investigated. The calibration curves of the 11 volatile PFC precursors showed good linearity in the concentration range of 10-500 μg/L with correlation coefficients not less than 0.9984. The limits of detection were 0.002-0.04 mg/kg (*S/N*=3), and limits of quantification were 0.006-0.1 mg/kg (*S/N*=10). The recoveries for the 11 analytes in different textile matrix samples at three spiked levels ranged from 73.2% to 117.2% with relative standard deviations (RSDs) of 0.1%-9.4% (*n*=6). Through actual sample analysis, four PFC precursors were detected in the textile product samples. The method has the advantages of simple pretreatment, accurate qualitative and quantitative analysis, high sensitivity, and good reproducibility. It can be effectively used for the simultaneous determination of 11 volatile PFC precursors in textiles. The establishment of this method has theoretical and practical significance for controlling PFC precursor levels in textiles. This study offers a new testing method for mitigating risk to safety and controlling textile products. It also provides a reference for establishing testing standards for PFC precursors in textiles and other similar consumer goods.

全氟化合物(perfluorinated compounds, PFCs)是碳氢化合物中的氢原子全部被氟原子取代后所形成的有机化合物,是一类持久性有机污染物^[1]^。PFCs的氟烷基链分子极性低,C-F键短,键能很大,因此PFCs具有优良的热稳定性、化学稳定性、高表面活性及疏水疏油性能,被作为整理剂和表面活性剂在纺织品的生产中大量使用^[2]^。

近年来,国内外对PFCs及其前体物的关注度越来越高,欧盟、加拿大、丹麦及美国各州纷纷出台相应政令或法案对PFCs及其前体物进行限制,且被限制使用的化合物种类有逐渐增多的趋势。国际环保纺织协会发布的生态纺织品标准(OEKO-TEX Standard 100)从2017年版起持续对氟调聚物醇(fluorotelomer alcohols, FTOHs)、氟调聚丙烯酸酯(fluorotelomer acrylates, FTAs)、全氟辛基磺酰胺(fluorooctane sulfonamides, FOSAs)和全氟辛基磺酰胺乙醇(fluorooctane sulfonamide ethanols, FOSEs)类化合物提出限量要求。2020年版的OEKO-TEX Standard 100对生态纺织品的限量要求为:包括2种FOSAs和2种FOSEs在内的7种全氟化合物及其前体物的总量不能超过1.0 μg/m^2^;此外,对婴幼儿纺织品的要求更为严格,所限制使用的PFCs及其前体物种类达33种之多,其中包括本文所关注的4种FTOHs及3种FTAs,要求该7种化合物每种均不能超过0.5 mg/kg。经对比发现,我国新发布的标准GB/T 18885-2020《生态纺织品技术要求》也对这33种PFCs及其前体物进行了限制使用,对于本研究所涉及的11种PFCs前体物的限量值要求与2020年版OEKO-TEX Standard 100限量值要求一致。

研究表明,PFCs具有肝毒性、胚胎毒性、生殖毒性、神经毒性和致癌性等,能干扰内分泌,改变动物的本能行为,对人类特别是幼儿可能具有潜在的发育神经毒性^[3,4,5]^。FTOHs和FTAs类化合物为全氟羧酸的前体物;FOSAs为全氟辛基磺酸的前体物^[6,7]^。摄入体内的PFCs前体物通过体内转化最终氧化生成全氟羧酸和全氟烷基磺酸,进一步威胁人类健康和生态安全。鉴于当前应用于纺织品等消费品中的PFCs前体物种类较多、多种前体物同时检测的方法还不成熟,对提升检测分析效能提出了更高的要求,因此,建立多种PFCs前体物同时检测的高效方法,对于我国纺织品等消费品中PFCs前体物检测标准的制定和产品质量安全风险管控,都具有理论和现实意义。

目前,国内外已有许多关于多种全氟化合物同时检测的报道^[8,9]^,多数方法采用液相色谱-串联质谱(LC-MS/MS)^[10,11,12,13,14,15,16,17]^,气相色谱-质谱(GC-MS)则相对较少^[17,18,19,20]^。随着对PFCs前体物研究的深入,对PFCs前体物的检测研究也十分活跃。Martin等^[21]^利用GC-MS法,将正、负化学源用于分析空气中7种PFCs前体物;罗建波等^[22]^采用气相色谱-正化学电离源质谱法建立了水和沉积物中7种PFCs前体物的测定方法;石瑀等^[23]^、杨琳等^[24,25]^则将LC-MS/MS法分别应用于血清^[23]^、母乳^[24]^和牛奶^[25]^等生物样品中PFCs前体物的含量测定;郭萌萌等^[26]^利用液相色谱-四极杆/静电场轨道阱高分辨质谱建立了鱼肉中18种PFCs及其21种前体物质的同时分析方法;张明等^[27]^和陈勇杰等^[28]^采用LC-MS/MS法,分别建立了大气降水^[27]^及污水与污泥基质^[28]^等环境样品中PFCs前体物的检测方法;张子豪等^[29]^采用气相色谱-串联质谱(GC-MS/MS)法建立了纸制食品接触材料中9种PFCs前体物的迁移量检测方法。用GC-MS法测定纺织品中PFCs前体物的文献有少数报道^[30,31]^,如程群等^[30]^采用GC-MS法测定纺织品中两种FOSEs,但两种化合物的检出限较高,为1.0 mg/kg,方法的应用受到一定限制。当前,将GC-MS/MS法用于纺织品中多种PFCs前体物的检测方法尚未见报道。基于此,本研究拟开发一种高效准确的测试方法,将气相色谱-串联质谱技术应用于纺织品中多种挥发性PFCs前体物的同时检测,也为建立完善的纺织品中PFCs前体物检测标准提供参考。

## 1 实验部分

### 1.1 仪器、试剂与材料

Trace1310-TSQ 9000气相色谱-三重四极杆质谱仪(美国Thermo Fisher Scientific公司), J&W VF-WAXms毛细管色谱柱(30 m×0.25 mm×0.25 μm)(美国Agilent公司), SK2510LHC超声波清洗仪(上海科导)。

标准品:1*H*,1*H*,2*H*,2*H*-全氟-1-己醇(4:2FTOH, 98.0%)、1*H*,1*H*,2*H*,2*H*-全氟-1-辛醇(6:2FTOH, 98.0%)、1*H*,1*H*,2*H*,2*H*-全氟-1-癸醇(8:2FTOH, 98.0%)、1*H*,1*H*,2*H*,2*H*-全氟-1-十二烷醇(10:2FTOH, 97.0%)、1*H*,1*H*,2*H*,2*H*-全氟辛基丙烯酸酯(6:2FTA, 97.0%)、1*H*,1*H*,2*H*,2*H*-全氟癸基丙烯酸酯(8:2FTA, 97.0%)、1*H*,1*H*,2*H*,2*H*-全氟十二烷基丙烯酸酯(10:2FTA, 98.0%)、*N*-甲基全氟辛烷磺酰胺(*N*-Me-FOSA, 97.0%)、*N*-乙基全氟辛烷磺酰胺(*N*-Et-FOSA, 98.0%)、*N*-甲基全氟辛烷磺酰胺乙醇(*N*-Me-FOSE, 95.0%)、*N*-乙基全氟辛烷磺酰胺乙醇(*N*-Et-FOSE, 95.0%),均购自加拿大Toronto Research Chemicals。本实验所用试剂均为色谱纯。11种全氟化合物前体物标准品的化学结构式见图1。

**图1:**
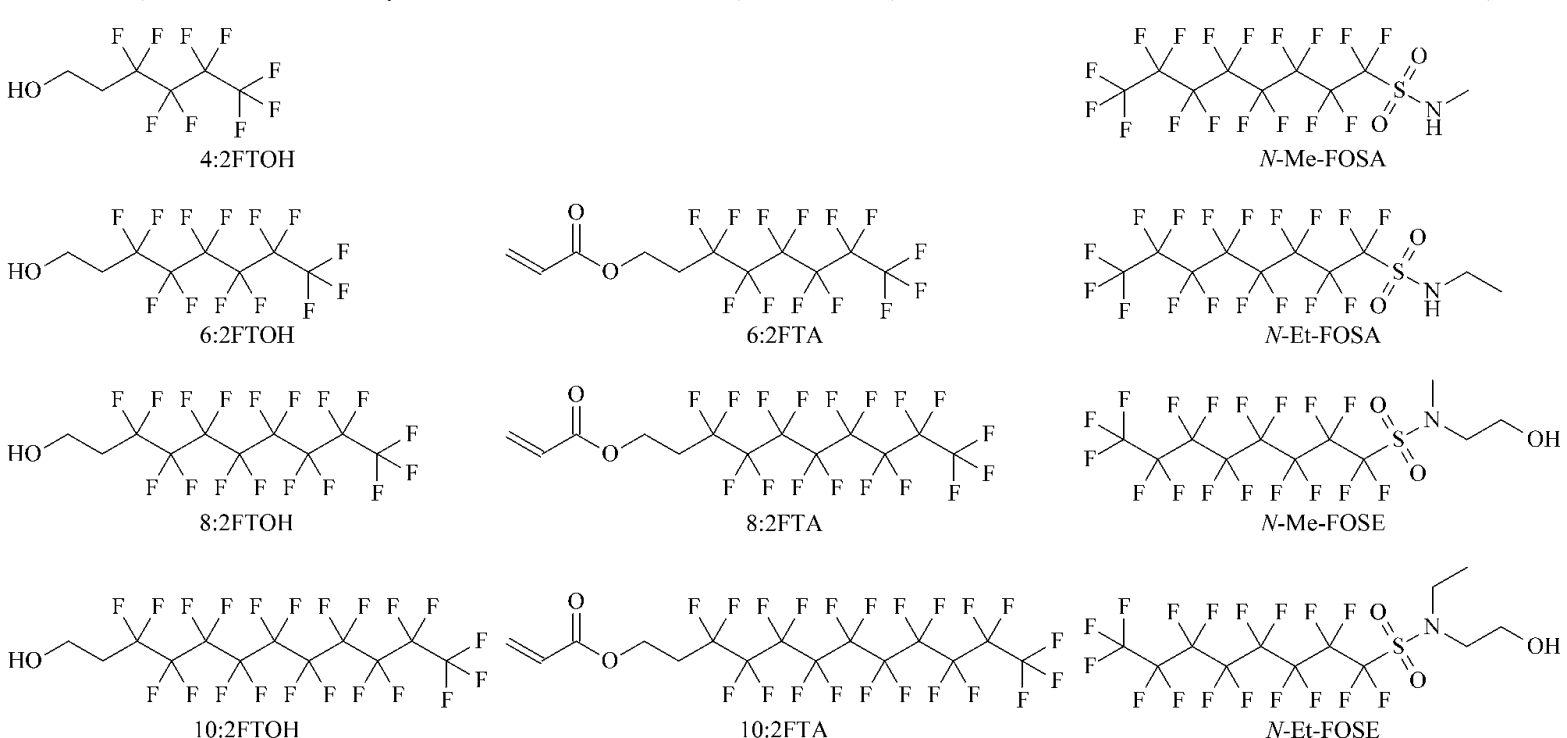
11种全氟化合物前体物的化学结构

### 1.2 标准溶液的配制

分别准确称取适量的11种全氟化合物前体物标准物质,用甲醇配制成200 mg/L的标准储备溶液。分别移取适量各单种标准储备溶液于适当体积的容量瓶中,用甲醇定容,配制成质量浓度为2.0、10和80 mg/L的混合标准中间溶液,于4 ℃冷藏保存。分别移取适量标准中间溶液于25 mL容量瓶中,用甲醇定容,配制成质量浓度分别为10、20、50、100、200、500 μg/L的系列混合标准工作溶液。

### 1.3 阳性样品的制备

取本实验室获得的阳性样品(含2种全氟化合物前体物的纺织布片),用碎布机切碎成5 mm×5 mm大小的碎片,混匀后用含有其他混合标准物质的溶液充分浸泡,期间每12 h充分搅拌一次,72 h后滤出,晾干,混匀,密封保存备用。

### 1.4 样品前处理

取代表性纺织品,切碎或剪碎成5 mm×5 mm的碎片,准确称取1.00 g试样于提取器中,加入20.0 mL甲醇,密封,于70 ℃水浴中超声60 min,冷却至室温,提取液经0.45 μm针式尼龙膜过滤后,按仪器分析条件进行测定。

### 1.5 气相色谱-串联质谱条件

气相色谱 高纯氦气为载气,柱流速为1 mL/min,进样口温度220 ℃,脉冲不分流进样,进样量2 μL,进样脉冲压力120 kPa,脉冲时间1 min,分流出口的吹扫流量100 mL/min,吹扫时间2 min,色谱柱升温程序为:初温40 ℃(保持1 min),以5 ℃/min升温至75 ℃(保持1 min),再以15 ℃/min升至240 ℃(保持4 min)。

质谱 电子轰击离子源(EI)温度250 ℃,传输线温度250 ℃,电离能量70 eV,采集模式:多反应监测离子模式(MRM), time模式。

## 2 结果和讨论

### 2.1 质谱条件优化

基于GC-MS/MS MRM模式可以将母离子和子离子一一对应的高选择性,通过设定多个时间段和扫描通道实现对纺织样品中11种全氟化合物前体物的同时检测。为了获得最佳的质谱条件保证对分析物定性和定量的准确性,对待测物的母离子、子离子和碰撞能量等参数进行考察。先通过GC分离和单级全扫描获得每种前体物的保留时间和一级碎片离子,选择响应较高的一级碎片离子为母离子,然后采用子离子扫描方式通过优化碰撞能量获得子离子,最后采用MRM模式对待测物进行定性和定量分析。试验选择的质谱参数见表1。

**表1:** 11种全氟化合物前体物的保留时间、监测离子对及碰撞能量

Compound	CAS No.	t/ min	MRM ion pairs (m/z)		CEs/ eV
6:2FTA	17527-29-6	7.31	55.0>27.0^*^;	418.1>99.0	25;42
4:2FTOH	2043-47-2	8.05	95.0>69.0^*^;	196.1>127.0	25;18
8:2FTA	27905-45-9	8.87	55.0>27.0^*^;	518.1>99.0	18;48
6:2FTOH	647-42-7	9.01	95.0>69.0^*^;	296.1>127.0	25;35
8:2FTOH	678-39-7	10.18	95.0>69.0^*^;	131.0>68.9	28;46
10:2FTA	17741-60-5	10.76	55.0>27.0^*^;	618.0>99.1	20;58
10:2FTOH	865-86-1	11.35	95.0>69.0^*^;	131.0>68.9	30;42
N-Et-FOSA	4151-50-2	15.75	108.0>80.0^*^;	131.0>68.9	10;44
N-Me-FOSA	31506-32-8	16.23	94.0>30.0^*^;	131.0>68.9	22;48
N-Me-FOSE	24448-09-7	17.30	526.0>462.0^*^;	131.0>68.9	52;39
N-Et-FOSE	1691-99-2	17.36	540.0>169.0^*^;	131.0>68.9	41;39

*Quantitative ion. CEs: collision energies.

图2是11种全氟化合物前体物混合标准溶液在已建立的条件下获得的MRM谱图,从图中可以看出11种化合物色谱峰分离较好,峰形对称且尖锐,能满足测试要求。

**图2:**
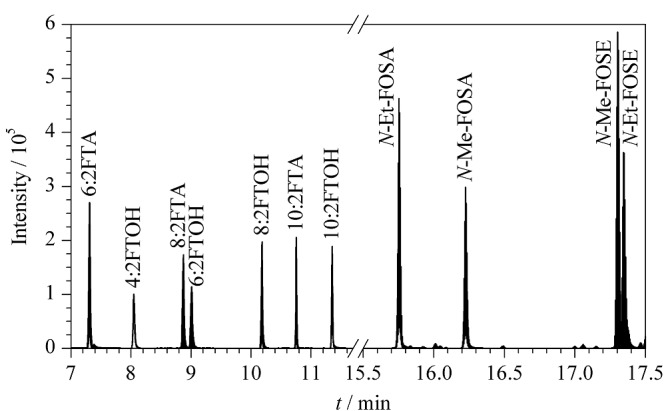
11种全氟化合物前体物的总离子流色谱图

### 2.2 样品处理条件的优化

2.2.1 提取溶剂的选择

提取是指通过溶解、吸着、挥发等方式将样品中的痕量全氟化合物前体物分离出来的操作步骤。由于全氟化合物前体物含量甚微,提取效率的高低直接影响分析结果的准确性,因此选择合适的提取溶剂至关重要。比较了几种常用的超声提取溶剂,甲醇、二氯甲烷、乙酸乙酯、正己烷、丙酮、乙腈的提取效果,甲醇、乙酸乙酯、乙腈作溶剂时色谱峰形较好,正己烷、二氯甲烷作溶剂时色谱峰较宽,丙酮作溶剂时出现杂峰,对出峰保留时间靠前的化合物形成干扰;相同分析条件下,目标物峰面积响应从高到低依次为:乙酸乙酯>甲醇>乙腈。与乙酸乙酯相比,甲醇作溶剂目标物的色谱峰更窄;又因为采用乙酸乙酯提取时提取液颜色较深,可能提取更多的杂质;且甲醇也常被用作纺织物中全氟化合物及其前体物的提取溶剂^[13,30,31]^,综合考虑,选择甲醇作为提取溶剂。

2.2.2 提取温度的优化

采用单因素变量法考察超声提取温度对11种全氟化合物前体物提取效率的影响,分别考察了40、50、60、70 ℃(常温常压下甲醇的沸点为64.7 ℃)条件下超声提取的效率,如图3所示,3种FTAs提取率随提取温度变化不明显;4种FTOHs提取率随提取温度的升高有增加的趋势,当提取温度为70 ℃, 4种FTOHs提取率最高;*N*-Et-FOSA、*N*-Me-FOSA、*N*-Me-FOSE和*N*-Et-FOSE 4种前体物提取率对提取温度的变化趋势不明显,在60 ℃条件下略高,但综合考虑,70 ℃条件下11种全氟化合物前体物相对提取效率均超过88.2%,故选择70 ℃为最终提取温度。

**图3:**
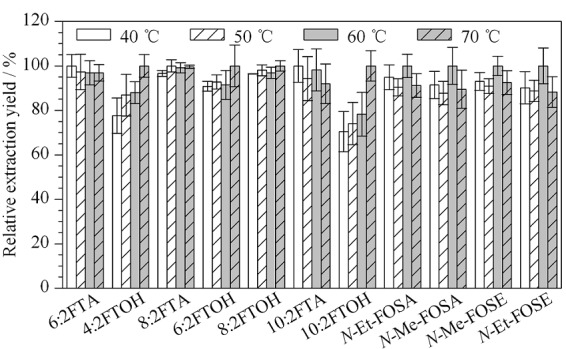
不同提取温度下11种全氟化合物前体物的提取 效率(*n*=3)

2.2.3 提取时间的优化

在提取温度为70 ℃条件下,比较了超声时间对全氟化合物前体物提取效率的影响,结果如图4所示,当提取60 min时大部分全氟化合物前体物基本提取完全,且继续增加提取时间,部分化合物提取量有所下降,因此超声提取时间选择60 min较为合适。

**图4:**
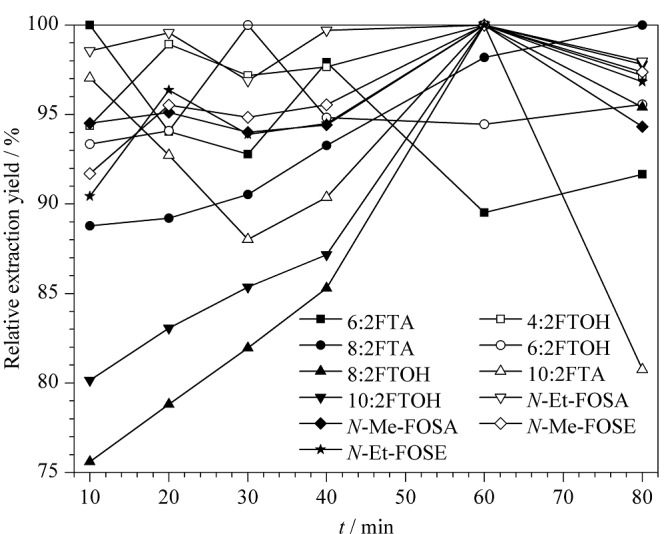
不同提取时间下11种全氟化合物前体物的提取效率

**图5:**
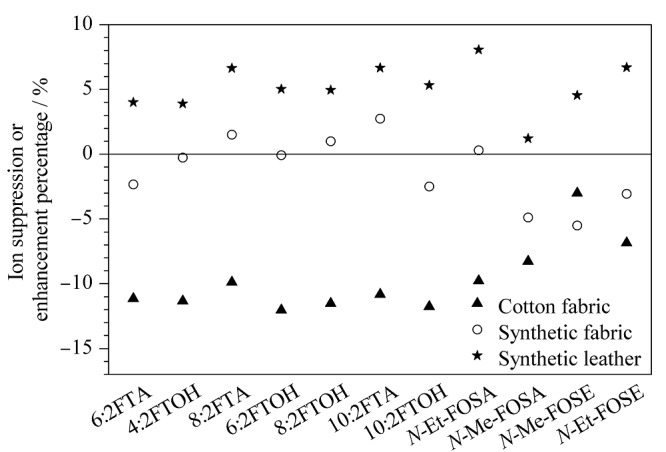
11种全氟化合物前体物的离子抑制或增强率

### 2.3 基质效应的考察

基质效应是指目标物以外的其他组分的存在对目标物测定值的影响,包括基质抑制和基质增强两种效应,用离子基质抑制率或增强率(*L*)表示^[32]^。*L*=(基质匹配标准曲线的斜率-溶剂标准曲线的斜率)/溶剂标准曲线的斜率×100%,当*L*>0,表示基质增强效应;当*L*<0,表示基质抑制效应。实验选取纯棉织物、合成纤维、合成革3种样品,考察11种全氟化合物前体物在实际样品中的基质效应,得到基质效应分布结果见图5。结果发现,不同材质的纺织基体对目标离子的作用有所不同,在合成纤维中的基质效应较弱,在纯棉织物基质中表现为一定的基质抑制效应,但在合成革基质中表现为一定的基质增强效应。11种前体物在3种基质的样品中*L*处于-12.0%~8.1%之间,说明本实验中的基质效应并不明显,实验结果可以接受。

### 2.4 标准曲线、检出限和定量限

用甲醇将标准储备液进行逐级稀释,配制成不同浓度的混合标准溶液,按照已建立的GC-MS/MS条件进行测定。结果发现11种全氟化合物前体物质量浓度在10~500 μg/L范围内,其峰面积*Y*与质量浓度*X* (μg/L)有良好的线性关系,线性相关系数*r* ≥ 0.9984。以信噪比为3(*S/N*=3)计算方法的检出限,以*S/N*=10计算方法的定量限, 11种全氟化合物前体物的线性方程、线性相关系数、检出限和定量限见表2。

**表2:** 11种全氟化合物前体物的线性方程、相关系数、 检出限和定量限

Compound	Regression equation	r	LOD/ (mg/kg)	LOQ/ (mg/kg)
6:2FTA	Y=1514X-7845	0.9996	0.02	0.06
4:2FTOH	Y=632.5X-3116	0.9992	0.01	0.04
8:2FTA	Y=1062X-3652	0.9991	0.03	0.01
6:2FTOH	Y=812.3X-4550	0.9991	0.01	0.03
8:2FTOH	Y=740.1X-1409	0.9987	0.002	0.006
10:2FTA	Y=833.2X-5348	0.9989	0.04	0.1
10:2FTOH	Y=642X-3177	0.9987	0.002	0.006
N-Et-FOSA	Y=1923X-12060	0.9986	0.004	0.01
N-Me-FOSA	Y=1179X-6168	0.9990	0.002	0.006
N-Me-FOSE	Y=2493X-17390	0.9987	0.003	0.01
N-Et-FOSE	Y=1557X-12250	0.9984	0.005	0.02

*Y*: peak area of the analyte; *X*: mass concentration, μg/L.

### 2.5 回收率和精密度

在纯棉织物、合成纤维和合成革3种材质的纺织样品中各分别添加100 μL 2.0、10和80 mg/L的混合标准溶液(添加水平相当于0.2、1和8 μg),按1.4节进行前处理,然后在GC-MS/MS实验条件下进样分析。每个添加水平平行做6个样品,计算11种全氟化合物前体物的平均回收率和相对标准偏差(RSD)。由表3可以看出,不同材质的纺织品中11种挥发性全氟化合物前体物在3个添加水平下的加标回收率为73.2%~117.2%, RSD为0.1%~9.4% (*n*=6),说明建立的分析方法准确可靠。

**表3:** 3种材质的纺织品样品中添加11种全氟化合物前体物的回收率及其相对标准偏差(*n*=6)

Compound	Recoveries/% (RSDs/%)											
	Cotton fabric				Synthetic fabric				Synthetic leather			
	0.2 μg	1 μg	8 μg		0.2 μg	1 μg	8 μg		0.2 μg	1 μg	8 μg	
6:2FTA	85.2 (8.3)	78.4 (6.3)	77.3 (5.4)		99.6 (6.2)	80.7 (6.4)	75.5 (6.2)		81.7 (4.5)	75.8 (6.7)	87.3 (9.3)
4:2FTOH	99.8 (5.2)	99.8 (3.8)	112.6 (8.4)		98.1 (5.6)	96.0 (1.3)	95.2 (2.0)		93.7 (7.4)	90.7 (1.6)	94.4 (6.0)
8:2FTA	114.0 (3.5)	115.2 (1.2)	114.6 (6.4)		108.3 (7.0)	115.3 (9.1)	105.9 (3.2)		73.2 (5.4)	81.5 (1.7)	76.0 (5.2)
6:2FTOH	105.2 (3.5)	99.6 (2.5)	103.3 (1.5)		100.7 (1.4)	95.7 (2.4)	93.2 (1.3)		100.6 (1.7)	117.2 (9.2)	101.0 (2.0)
8:2FTOH	106.5 (2.1)	94.4 (5.8)	105.4 (3.6)		111.7 (4.3)	90.6 (4.6)	93.5 (2.1)		92.5 (4.4)	95.4 (4.2)	101.3 (5.0)
10:2FTA	99.7 (3.8)	97.3 (3.7)	102.6 (5.9)		101.8 (3.8)	100.9 (4.1)	101.3 (3.3)		95.6 (2.5)	98.6 (3.0)	98.0 (5.2)
10:2FTOH	116.2 (4.0)	96.3 (6.5)	100.1 (4.0)		99.1 (0.1)	90.8 (5.4)	90.4 (3.5)		78.8 (4.1)	86.5 (3.7)	99.3 (5.4)
N-Et-FOSA	92.8 (7.8)	84.7 (6.3)	91.0 (8.7)		95.3 (7.0)	81.3 (5.6)	90.2 (3.4)		105.9 (8.2)	92.5 (7.3)	98.5 (1.0)
N-Me-FOSA	94.5 (7.4)	80.5 (6.3)	87.4 (6.2)		86.6 (9.0)	79.4 (5.9)	91.7 (4.8)		96.4 (7.0)	90.3 (4.7)	101.9 (2.6)
N-Me-FOSE	114.6 (3.3)	102.5 (7.7)	104.8 (7.9)		95.1 (8.5)	93.0 (7.6)	99.6 (8.9)		110.1 (6.3)	94.5 (9.2)	100.5 (9.4)
N-Et-FOSE	103.6 (6.5)	102.3 (8.3)	105.9 (9.1)		100.5 (5.5)	97.4 (9.2)	98.7 (8.9)		107.4 (8.7)	97.8 (4.2)	101.8 (5.8)

### 2.6 实际样品测试

在纺织品检测中发现,涂层纺织品(经涂层整理的纺织品)往往被列为全氟化合物测试项目的重点考核对象之一。采用本方法对30个纺织样品进行检测,包括10个非涂层纺织品、10个涂层纺织品和10个合成革。当样品检出浓度较低时(如2^#^样品),根据预测的浓度值,配制相近浓度标准溶液进行分析,以单点外标法对样品重新进行定量,结果如表4所示,所检测的30个测试样品中,有13个样品检出6:2FTOH、8:2FTOH、10:2FTOH以及*N*-Me-FOSE等前体物,涂层纺织品和非涂层纺织品均可能存在全氟化合物前体物;所测试的10个合成革样品有7个检出FTOHs,检出概率较大,且检出8:2FTOH和10:2FTOH含量较高。

**表4:** 纺织样品中11种全氟化合物前体物的分析结果(仅列出有检出目标物的样品)

Compound	Contents/(mg/kg)																								
	Uncoated textile samples				Coated textile samples				Synthetic leather samples																
	1^#^	2^#^	3^#^		4^#^	5^#^	6^#^		7^#^	8^#^	9^#^	10^#^	11^#^	12^#^	13^#^										
6:2FTA	-	-	-		-	-	-		-	-	-	-	-	-	-
4:2FTOH	-	-	-		-	-	-		-	-	-	-	-	-	-
8:2FTA	-	-	-		-	-	-		-	-	-	-	-	-	-
6:2FTOH	-	-	26.3		0.38	-	-		-	3.63	6.72	3.68	3.81	3.34	2.94
8:2FTOH	0.24	-	-		0.72	0.61	26.2		196	1058	1745	1398	1269	1421	347
10:2FTA	-	-	-		-	-	-		-	-	-	-	-	-	-
10:2FTOH	-	-	-		0.34	2.83	100		41.1	327	572	497	405	1837	386
N-Et-FOSA	-	-	-		-	-	-		-	-	-	-	-	-	-
N-Me-FOSA	-	-	-		-	-	-		-	-	-	-	-	-	-
N-Me-FOSE	-	0.02	-		-	-	-		-	-	-	-	-	-	-
N-Et-FOSE	-	-	-		-	-	-		-	-	-	-	-	-	-

-: not detected.

## 3 结论

本研究利用GC-MS/MS技术,建立了一种高效且能同时检测纺织品中11种全氟化合物前体物的分析方法。该方法快速简便、耗时短,具有良好的灵敏度、准确度和精密度,为纺织品全氟化合物前体物风险监控提供了一种快速、高效、可靠的分析手段。通过实际样品测试分析发现,全氟化合物前体物已然被较广泛地用于国内纺织品整理之中。该法可为当前相关产品安全质量风险防控提供参考。
